# Formulation and characterization of solid dispersion adsorbate of Candesartan Cilexetil: in vitro dissolution and in vivo performance

**DOI:** 10.3389/fddev.2026.1975209

**Published:** 2026-10-26

**Authors:** Harshad Makwana, Anjali Patel, Manisha Lalan, Meenakshi Patel, Nirmal Shah, Bhavin Vyas, Bhupendra Prajapati, Pranav Shah

**Affiliations:** 1 Maliba Pharmacy College, Surat, India; 2 Parul University, Vadodara, India; 3 Sumandeep Vidyapeeth Deemed to be University, Vadodara, India; 4 Uka Tarsadia University, Surat, India

**Keywords:** 3^2^ factorial design, bioavailability, candesartan cilexetil, hypertension, solid dispersion adsorbate

## Abstract

**Introduction:**

Candesartan Cilexetil (CC) is a non-peptide angiotensin II type 1 receptor antagonist widely used in the treatment of hypertension. However, CC has poor aqueous solubility (0.006 ± 0.002 mg/mL), low oral bioavailability (15%) and undergoes significant first-pass metabolism.

**Methods:**

Candesartan Cilexetil Solid Dispersion adsorbate (CC-SDAs) was formulated by the fusion method, employing polyethylene glycol (PEG) 4000 as a hydrophilic carrier and Neusilin® US2 as an adsorbent, to improve the solubility, dissolution, and oral bioavailability of CC. The formulation was optimized using a 3^2^ factorial design.

**Results and Discussion:**

The optimized SDA formulation exhibited the following properties: Carr’s index (9.86 ± 0.09%), Hausner’s ratio (1.10 ± 0.16) and angle of repose (26.00 ± 0.19°), which shows improved flow property. DSC and XRD studies confirmed the conversion of the crystalline drug into an amorphous form. The optimized SDA formulation achieved %CDR of 90.41 ± 0.95% at Q30 min, demonstrating a marked improvement in in vitro dissolution compared to the pure drug (%CDR of 57.20 ± 3.78% at Q30 min). Furthermore, in vivo pharmacokinetic studies revealed a 2.02-fold enhanced oral bioavailability of CC from the SDA formulation compared to the CC suspension. Therefore, the SDA technique proves to be a promising approach for enhancing the solubility and dissolution of poorly water-soluble drugs.

## 1 Introduction

High blood pressure, commonly known as hypertension, occurs when the force of flowing blood against the arteries is constantly elevated (130/80 mmHg). For cardiovascular disease (CVD), hypertension is an important and avoidable risk factor (Dixit and Dhakad, 2017). In 2024, an estimated 1.4 billion people had hypertension (World Health Organization, 2025).

Hypertension is a major cause of early mortality and disability worldwide, accounting for around 10 million deaths annually (Liu et al., 2024). Hypertension is treated using drugs like Candesartan Cilexetil (CC), Olmesartan, losartan, telmisartan and valsartan (Carey et al., 2022). Clinical data demonstrated that Candesartan Cilexetil is more effective than losartan for lowering blood pressure due to its minimal metabolism requirement and its potential for use in patients with mild hepatic dysfunction and is at least as effective as telmisartan, and valsartan (Cernes et al., 2011). Candesartan Cilexetil is a non-peptide angiotensin II type 1 receptor antagonist, and it is used to treat heart failure and hypertension (Aparna et al., 2023). Due to its poor solubility (0.006 ± 0.002 mg/mL) and incomplete dissolution in the gastrointestinal fluids, it is a BCS class II drug with 15% oral bioavailability (Jain et al., 2016). It is a lipophilic drug (log P is 6.1) with low molecular weight (Mw: 610.67 g/mol) (Nekkanti et al., 2009). Candesartan Cilexetil is now available as a non-modified release tablet in doses of 2, 4, 16, and 32 mg (Khairnar et al., 2012).

The reported attempts to enhance the bioavailability of Candesartan Cilexetil have explored the use of techniques such as self-nanoemulsifying drug delivery systems (SNEDDS) (Aboul Fotouh et al., 2019), solid lipid nanoparticles (SLN) (Dudhipala and Veerabrahma, 2016), nanostructured lipid carriers (NLCs) (Anwar et al., 2020), novel mucoadhesive buccal films (Mady et al., 2021), and niosomes coated with chitosan (AbuElfadl et al., 2021). Although these methods have demonstrated varied degrees of increased bioavailability, they are also expensive. Consequently, a method that is economical, efficient, and scalable in the industrial setting is required. The solid dispersion adsorbate (SDAs) approach offers all of these benefits in addition to being a green process choice (Yadav et al., 2021). SDAs are formed by solvent evaporation, or melting, or melting-solvent techniques to disperse the active agent or components into an inert carrier. SDA methods facilitate solid dispersion (SD), which can physically adsorb to the surfaces of inert porous materials. Combining SD and melt adsorption technology improves drug solubility through SD and increases the drug’s effective surface area (Arunkumar et al., 2009). SDA is one of the most widely used methods for improving the bioavailability of weakly water-soluble actives due to its industrial viability and affordability.

We attempted to formulate SDAs in which PEG 4000 was used as a hydrophilic carrier and Neusilin® US2 was used as an adsorbate, which improved Candesartan Cilexetil dissolution (Yadav et al., 2021; Tian et al., 2014; Mujtaba et al., 2025). PEG 4000 possesses high aqueous solubility, a low melting point, and excellent wetting properties that facilitate the formation of a molecularly dispersed system during the fusion process. Neusilin® US2 has an exceptionally high specific surface area, high porosity, and excellent adsorption capacity, which allow efficient loading of melted SD onto its porous structure without compromising flow or compressibility (Mesnukul et al., 2009; Vidiyala et al., 2025). This fusion method makes the dosage form both safe and affordable by eliminating the use of toxic solvents (Kim and Chen, 2018).

In the current investigation, CC-SDAs were formulated using a technique to increase solubility and dissolution and to transform them into granules that are compressible and flowable. A design of experiments (DoE) approach with a 3^2^ factorial design was applied to optimize CC-SDAs. This allowed for the analysis of how the independent variables, such as the amount of carrier (PEG 4000) (X_1_) and the amount of adsorbate (Neusilin® US2) (X_2_), affect the dependent variables, % cumulative drug release (%CDR) Q30 min (Y_1_) and Carr’s index (Y_2_). The optimized formulation was evaluated using FTIR, DSC, XRD, SEM, saturated solubility, drug content, an *in vitro* dissolution study, and an *in vivo* pharmacokinetic study.

## 2 Materials and methods

### 2.1 Materials

A gift sample of Candesartan Cilexetil was provided by Alembic Pharma Pvt. Ltd., Vadodara, India. Tween® 20, methanol, potassium dihydrogen phosphate, and potassium bromide (KBr) were obtained from Loba Chemicals Pvt. Ltd., Mumbai, India. PEG 4000 was acquired from S.D. Fine Chemical Products, Mumbai, India. Neusilin® US2 was bought from Gangwal Chemicals Pvt. Ltd., Mumbai, India. All samples are of analytical grade.

### 2.2 Methods

#### 2.2.1 Preparation of CC-SDAs

The SDA of Candesartan Cilexetil was formulated using the fusion technique. This technique employed different amounts of Neusilin® US2 as an adsorbate (32 mg, 96 mg, and 160 mg) and different amounts of PEG 4000 (32 mg, 96 mg, and 160 mg). To formulate the CC-SDA, Candesartan Cilexetil was added to melted PEG 4000 by heating up to its melting point (57 °C–63 °C) in a porcelain dish set over a water bath at 60 °C and stirred manually with the glass rod for 3 min. The adsorbate Neusilin® US2 was added, then the mixture was rotated rapidly until it became a blend. The mixture was then allowed to cool to room temperature. To create a uniformly sized, free-flowing powder, the SDAs were passed through a 40# sieve (Mujtaba et al., 2025; Sakur and Fael, 2010; Vaghela et al., 2021; Patel et al., 2013).

#### 2.2.2 Preparation of CC-Physical mixture (PM)

To formulate the physical mixture, 32 mg of Candesartan Cilexetil, 41.47 mg of PEG 4000, and 159.36 mg of Neusilin® US2 were simply combined for 10 min using a spatula (Yadav et al., 2021).

#### 2.2.3 Saturated solubility

The saturated solubility of Candesartan Cilexetil was estimated by adding an excess amount of Candesartan Cilexetil to glass vials containing 50 mL of distilled water. The mixture was then orbitally shaken (REMI CIS-18, Mumbai, India) for 24 h at 25 °C. After centrifuging the solution for 30 min at 10,000 rpm and 25 °C (REMI C24 plus, Vasai, India), the supernatant was collected and filtered through a 0.45 µm nylon syringe filter (Millipore, United States). The filtrate was diluted, and the absorbance was measured using a UV spectrophotometer (UV 3092, Lab India Analytical Instrument Pvt. Ltd., Thane, India) at λmax of 257 nm. The saturated solubility of the produced CC-SDAs was assessed using a similar method (Ravisankar et al., 2016).

#### 2.2.4 Drug content

Accurately weigh CC-SDAs equivalent to 32 mg of Candesartan Cilexetil and dissolve in 100 mL of methanol. The resulting solution was filtered, and then a proper dilution was done using methanol. Then the drug content was assessed using a UV spectrophotometer at λmax 257 nm against a blank (Kamalakkannan et al., 2013).

#### 2.2.5 Design of experiment (DoE)

DoE is a systematic approach to determining the relationships between independent and dependent variables in a process (ICH, 2009b). To gather as much information as possible, experiments are designed and executed. Key components are altered through carefully monitored experiments, and the results are then correlated using a mathematical model. The various statistical parameters were used to assess and choose the best-fitted model, including coefficient of determination (R^2^), adjusted R^2^, predicted R^2^, coefficient of variation (C.V.), standard deviation, predicted residual sum of squares (PRESS), lack-of-fit, and regression data. A three-dimensional response surface graph was also created for every response using Design Expert® Software Version 11.0 (STAT Ease, Inc., United States) (Vaghela et al., 2021; Mahajan et al., 2018). According to several studies, the amount of carrier and the amount of adsorbent affect the *in vitro* dissolution study and physicochemical properties of SDAs (Mahajan et al., 2018; Vojinović et al., 2018; Gupta et al., 2002). The formulation was optimized using a 3^2^ factorial design with both independent and dependent variables. The amount of carrier (PEG 4000) (X_1_) and the amount of adsorbate (Neusilin® US2) (X_2_) were the independent variables, while %CDR at Q30 min (Y_1_) and Carr’s Index (Y_2_) were dependent variables (Kumria et al., 2018), as displayed in Table 1. The trial run composition that the software recommends is shown in Table 2.

**TABLE 1:** Independent and dependent variables and their selected levels for the formulation of SDAs.

Independent variables			
Levels	Low (−1)	Medium (0)	High (+1)
Factors			
Amount of carrier (PEG 4000) (X_1_) (mg)	32	96	160
Amount of adsorbate (neusilin® US2) (X_2_) (mg)	32	96	160

Dependent variables		Constraints	
Y_1_-%CDR at Q30 min		Not less than (NLT) = 80%	
Y_2_- Carr’s index (%)		Not more than (NMT) = 10%	

**TABLE 2:** Compositions of SDAs batches according to design.

Batch no.	Amount of carrier (PEG 4000) (X_1_)	Amount of adsorbate (neusilin® US2) (X_2_)
B_1_	160	96
B_2_	32	96
B_3_	96	160
B_4_	96	96
B_5_	96	32
B_6_	32	160
B_7_	32	32
B_8_	160	32
B_9_*	96	96
B_10_*	96	96
B_11_	160	160
B_12_*	96	96

(* = center point batches).

After evaluating the main impacts, interaction effects, and quadratic effects of the formulation ingredients on flow characteristics and *in vitro* release of formulations, a two-factor, three-level design was utilized to statistically optimize the formulation parameters. The quadratic model that the computer generated is provided as

$$Y=b_{0}+b_{1}X_{1}+b_{2}X_{2}+b_{12}X_{1}X_{2}+b_{11}X_{1}^{2}+b_{22}X_{2}^{2}$$ (2.1)

The dependent variable is represented by the term Y. If b_0_ is the arithmetic average response of twelve different runs, b_1_ is an estimated coefficient for the independent variable X_1_, and b_2_ is an estimated coefficient for the independent variable X_2_. The dependent variable is represented by the term Y. The main effect (X_1_ and X_2_) shows the mean outcome of changing one independent variable at a time from its lower to a higher value. When the independent variables are changed simultaneously, the dependent variable changes as indicated by the interaction term (X_1_X_2_). To identify nonlinearity, polynomial terms (X_1_^2^ and X_2_^2^) have been introduced. The two dependent variables were Y_1_, %CDR at Q30 min, and Y_2_, Carr’s index %, at the low (−1), medium (0), and high (+1) levels (9 factorial +3 centre points), each with varying amounts of carrier (PEG 4000) = (X_1_) (32 mg, 96 mg, 160 mg) and amounts of adsorbate (Neusilin® US2) = (X_2_) (32 mg, 96 mg, 160 mg), were created for the experiment.

#### 2.2.6 Precompression evaluation of SDAs

##### 2.2.6.1 Compressibility and flowability

The compressibility of the CC-SDA mixture was evaluated using Carr’s index and Hausner’s ratio. The calculated quantity of the powder mixture was put into a measuring cylinder. Both the bulk volume and the tapped volume were measured. Numerous metrics were determined, including bulk density, tapped density, Hausner’s ratio, and Carr’s index (Kinoshita et al., 2002).

A fixed-height funnel technique was used to determine the angle of repose (θ), which allowed for the determination of the flow characteristics of the CC-SDAs blend. The purpose of the powder mixture was to flow out of the funnel until the mixture created at the tip of the conical pile slightly touched the funnel’s tip. A measure of the average (r) blends was taken. In order to calculate the angle of repose, the funnel’s height was maintained at 2 cm (Ganesan et al., 2008).

#### 2.2.7 Post-evaluation

##### 2.2.7.1 *In vitro* dissolution study

The dissolution profile of capsules (00-size gelatin capsules) filled with CC-SDA batches was done at 50 rpm using USP type II equipment (Electro Lab TDT-08L, Mumbai, India) (Kumar and Suresh, 2021). The 900 mL of 0.70% Tween® 20 in phosphate buffer pH 6.5 was employed as the dissolution medium, and the temperature was set to 37 °C ± 0.5 °C. 5 mL samples were withdrawn, and the same amount was added at 10, 20, 30, 45, and 60 min. The sample was filtered using a Millipore filter (0.45 μm) and then subjected to UV spectroscopy at 257 nm. At different time intervals, the cumulative percentage release of Candesartan Cilexetil was computed using the calibration curve formula. Similarly, *in vitro* dissolution profiles of optimized CC-SDA were compared with those of the pure drug and CC-PM (00-sized gelatin capsule) (Amer et al., 2018).

#### 2.2.8 Generation of polynomial equations and response surface plots

For the current CC-SDA research, the Design-Expert software was used to perform a number of response surface methodology (RSM) calculations. Polynomial models were developed for each response variable. 3D graphs were created using the output files produced by the Design-Expert program. Analysis of variance (ANOVA) was used to determine how significant these parameters were for the variables (Shah et al., 2021a).

### 2.3 Validation of experimental design

Check-point analysis was used to validate the 3^2^-factorial design used in the production of CC SDAs. An optimum batch of CC-SDAs was produced based on the values of independent variables obtained from the overlay plot. The correctness of the design was verified by comparing the predicted and experimental values of the responses (Gandhi et al., 2017).

#### 2.3.1 Characterization of optimized CC-SDAs

##### 2.3.1.1 Fourier transform infrared spectroscopy (FTIR)

A qualitative FTIR analysis (IR-470; Shimadzu, Japan) of Candesartan Cilexetil and optimized CC-SDAs was performed. Using an empty pellet container as a reference, 1–2 mg of the sample was weighed, mixed with KBr, compressed into discs in a compressor unit under vacuum, and scanned between 3500 and 1000 cm^-1^ (Kamalakkannan et al., 2013).

##### 2.3.1.2 Differential scanning calorimetry (DSC)

Approximately 5 mg of the Candesartan Cilexetil and optimized CC-SDAs were sealed in a crimped aluminum pan for analysis (DSC-60, Shimadzu, Japan). Before the analysis, the samples were desiccated over calcium chloride for a full day in an effort to eliminate surface-absorbed water. The thermograph was analyzed qualitatively by looking at the endothermic transition contour and the peak temperature. The heating rate was 5 °C per min between 40 °C and 340 °C, and the nitrogen flow rate was 20 mL per min (Tulojirao et al., 2023).

##### 2.3.1.3 Powder X-ray diffraction (XRD)

A powder X-ray diffractometer (PW 1710, Philips, Amsterdam, Netherlands), was utilized to analyze Candesartan Cilexetil and optimized CC-SDA’s diffraction pattern. Cu k-alpha radiation served as the X-ray source. For examination, samples were put in polystyrene glass sample holders. The sample holder was inserted into the X-ray diffractometer that had a scintillation counter detector installed. X-ray powder diffraction patterns in the 0°–50° range were obtained using a voltage of 45 kV, a current of 40 mA, and a scan angle of 2°/min (Tulojirao et al., 2023).

##### 2.3.1.4 Scanning electron microscopy (SEM)

A scanning electron microscope was used to analyze the layered sample’s surface morphology (SSX-550, Shimadzu, Kyoto, Japan) with an image analysis system (Image Inside version 2.32). The Candesartan Cilexetil and optimized CC-SDAs were imaged at different magnifications using an SEM, and the photos were directly captured on a computer. Analysis was performed at 25 °C ± 2 °C, and the image was captured on a scanning electron micrograph (accelerating voltage: 15.0 kV) (Kamalakkannan et al., 2013).

##### 2.3.1.5 Contact angle

A drop shape analyzer (Theta Lite Optical Tensiometer, Biolin Scientific, Germany) was used to measure the contact angle of Candesartan Cilexetil and CC-SDA samples using the sessile drop method. The sessile drop goniometric method was utilized to measure the contact angle with water as the polar liquid. An OCA-20 contact angle meter from Data Physics Instruments was used to measure the contact angle using SCA 20 software (version 3.4.6). A high-speed camera took pictures of the liquid drop 0.02 s after a tiny drop was placed on the disk using a tip. Every measurement was conducted at 25 °C ± 2 °C (Juppo et al., 2003).

##### 2.3.1.6 Sample extraction and bioanalysis

Accurately weighed 10 mg of Candesartan Cilexetil and added it to a separate 10 mL volumetric flask, and methanol was added up to the mark to achieve the final volume of 1000 μg/mL. From the above stock solution, aliquots of 0.1, 0.2, 0.3, 0.4, and 0.5 mL were accurately withdrawn with the help of a pipette and transferred to a separate 10 mL volumetric flask, and the volume was made up to the mark with methanol to get the final concentrations of 10, 20, 30, 40, and 50 μg/mL. From each solution, take 10 µL of solution and spike with 50 µL of plasma. Then the mixture was vortexed for 5 min. Add 1 mL of acetonitrile and then centrifuge for 15 min at 10,000 rpm and 4 °C. The resulting supernatants were taken and filtered, and the volume was made up to the mark with methanol to achieve the final concentrations of 10, 20, 30, 40, and 50 ng/mL. The fluorescence intensity of Candesartan Cilexetil solutions was assessed using a Shimadzu 5301 PC spectrofluorometer at an excitation wavelength of 260 nm and an emission wavelength of 381 nm (Kumar and Suresh, 2021).

##### 2.3.1.7 *In vivo* pharmacokinetic study

The animal experiment was conducted in accordance with the guidelines established by the Committee for Control and Supervision of Animal Experiments (CCSEA) of the Government of India. The animal protocol (MPC/IAEC/38/2024) was accepted by the Institutional Animal Ethics Committee (IAEC) of Maliba Pharmacy College, which was followed throughout the experiment.

Weighed male Wistar rats around 250 ± 16 g and kept them in a controlled environment with a 12-h light/dark cycle and a temperature range of 22 °C–26 °C. In this investigation, a total of eighteen rats were used. The animals were split into three groups. Each group received six rats. Animals in groups A, B, and C were kept fasting all night. 1 mL of drug suspension, CC-PM, and CC-SDA suspension was administrated. Drug suspension (dose equivalent to 3 mg/kg) was dispersed in 0.5% w/v sodium carboxymethyl cellulose) was given to Group A; in Group B, CC-PM was given (dose equivalent to 3 mg/kg), while Group C was given the optimized CC-SDA suspension (3 mg/kg). Blood was drawn from the retroorbital plexus at predetermined intervals (0 min, 15 min, 30 min, 1 h, 2 h, 3 h, 4 h, 6 h, 8 h, and 24 h). To extract the plasma, the obtained blood samples were centrifuged for 15 min at 4 °C and 10,000 rpm. After being separated from rat plasma, Candesartan Cilexetil was measured using the spectrofluorimetric method (Aparna et al., 2023).

##### 2.3.1.8 Stability studies

A stability study was conducted for 6 months in accordance with ICH recommendations. The final optimized CC-SDA was maintained at 40 °C ± 2 °C/75% ± 5% RH relative humidity in a closed glass vial within the stability chamber (Thermo Lab, India). At the end of 6 months, stability research was conducted to assess saturated solubility, %CDR at Q30 min, angle of repose, Carr’s index, and Hausner’s ratio (Shah et al., 2021b).

#### 2.3.2 Statistical analysis

Concentration–time data were analyzed using a linear mixed-effects model with formulation, time (categorical), and their interaction as fixed effects, and subject as a random intercept to account for repeated measurements within animals. When the formulation × time interaction was significant, pairwise formulation comparisons were performed separately at each time point using model-estimated contrasts. P-values for the three pairwise comparisons within each time point were adjusted by the Holm method. Tests were two-sided, and p < 0.05 was considered statistically significant. Exact p-values are reported.

## 3 Results and discussion

### 3.1 Saturated solubility

Prepared batches (B_1_–B_12_) were evaluated for saturated solubility, and the outcomes are presented in Table 3. A significant improvement in the saturated solubility of Candesartan Cilexetil was observed with the incorporation of carriers and adsorbates such as PEG 4000 and Neusilin® US2 (Yadav et al., 2021; Van den Mooter et al., 1998). The solubility of the optimized batch was achieved at 0.057 ± 0.005 mg/mL. This enhancement in solubility may be related to the hydrophilic nature of PEG 4000, which improves the wettability of the Candesartan Cilexetil, and to Neusilin® US2, which acts as an adsorbent and increases the surface area of the formulation (Chernetska et al., 2021; Solanki et al., 2025).

**TABLE 3:** Formulation table of CC-SDAs as per 3^2^ factorial designs.

Batches	%CDR at Q30 min (%) (Y_1_)	Carr’s index (%) (Y_2_)	Hausner’s ratio	Angle of repose (°)	Saturated solubility (mg/mL)	Drug content (%)
CC	57.20 ± 3.78	23.11 ± 0.48	1.30 ± 0.08	33.45 ± 0.43	0.0060 ± 0.002	99.45 ± 2.30
B_1_	82.40 ± 0.46	9.20 ± 0.39	1.20 ± 0.01	34.02 ± 1.52	0.0373 ± 0.002	95.36 ± 2.11
B_2_	77.35 ± 0.43	5.10 ± 0.73	1.14 ± 0.01	29.90 ± 0.52	0.0151 ± 0.004	95.13 ± 3.42
B_3_	97.30 ± 0.46	17.30 ± 0.90	1.15 ± 0.01	28.60 ± 1.83	0.0403 ± 0.001	95.56 ± 2.43
B_4_	85.40 ± 0.43	15.40 ± 0.29	1.12 ± 0.02	31.75 ± 0.95	0.0376 ± 0.002	95.85 ± 2.11
B_5_	89.40 ± 0.45	20.10 ± 0.55	1.25 ± 0.01	38.71 ± 1.34	0.0277 ± 0.002	96.38 ± 2.80
B_6_	86.33 ± 0.40	7.70 ± 0.51	1.11 ± 0.01	22.53 ± 2.99	0.0504 ± 0.004	98.35 ± 1.96
B_7_	80.40 ± 0.27	11.20 ± 0.97	1.23 ± 0.01	34.75 ± 0.63	0.0107 ± 0.004	96.81 ± 2.60
B_8_	82.55 ± 0.48	12.10 ± 0.37	1.26 ± 0.01	36.86 ± 0.65	0.0306 ± 0.002	96.39 ± 2.33
B_9*_	85.10 ± 0.30	15.03 ± 0.54	1.18 ± 0.03	31.94 ± 1.50	0.0306 ± 0.003	97.31 ± 3.32
B_10*_	86.94 ± 0.45	15.60 ± 0.41	1.19 ± 0.02	31.71 ± 1.11	0.0375 ± 0.003	95.85 ± 3.90
B_11_	92.98 ± 0.45	14.30 ± 0.10	1.15 ± 0.02	32.30 ± 1.00	0.0455 ± 0.004	96.30 ± 2.68
B_12*_	86.17 ± 0.32	14.60 ± 0.50	1.16 ± 0.04	33.14 ± 1.73	0.0345 ± 0.003	97.41 ± 1.76

(n = 3, ±SD), (* = center point batches).

### 3.2 Drug content

SDA’s drug content ranged from 95.13% ± 3.42% to 98.35% ± 1.96%. This outcome shows how well the fusion process works with the materials utilized for these kinds of preparations.

### 3.3 3^2^ factorial design

The interaction between the independent and dependent variables was investigated using the 3^2^-factorial design. Polynomial models (quadratic, two-factor interaction, and linear) were created using the software by adding the collected response values. Based on the R^2^ value, the quadratic model was determined to be the most appropriate model for Y_1_ and Y_2_. The quadratic models Y_1_ and Y_2_ have P values of <0.0001, indicating their significance.

#### 3.3.1 Data analysis of %CDR at Q30 min (Y_1_)

The *in vitro* release profile of CC-SDAs and pure drug was shown in Table 3. CC-SDAs exhibited % CDR (Q30 min) in the range of 77.35% ± 0.43% to 97.30% ± 0.46%. The model’s F value of 88.89 indicated its significance (Table 4). To determine how each independent variable affected the outcome, an ANOVA was conducted (Table 4). In this case, the model terms A, B, AB, A^2^ and B^2^ are significant (p < 0.05). The F-value of 1.13 (p = 0.4618) indicates that the lack of fit is not significant. A reasonable match was suggested by the correlation coefficient value (R^2^), which was found to be 0.9867 (Table 5). The adjusted R^2^ of 0.9756 (Table 5) was reasonably close to the predicted R^2^ value of 0.9403. An adequate signal was indicated by an adequate precision value of 33.20 (>4) (Table 5).

$$\%\text{CDR at }Q30\min\left(Y_{1}\right)=+86.18+2.31A+4.04B+1.13\text{AB}-6.90A^{2}+6.58B^{2}$$ (3.1)

**TABLE 4:** (A) ANOVA table of %CDR at Q30min (B) ANOVA table of Carr’s index.

(A) Response Y_1_						
Source	Sum of square	Df	Mean square	F-value	p-value	
Model	316.90	5	63.38	88.89	<0.0001	Significant
A-amount of carrier	31.97	1	31.97	44.84	0.0005	
B-amount of adsorbate	98.09	1	98.09	137.57	<0.0001	
AB	5.06	1	5.06	7.10	0.0373	
A^2^	126.87	1	126.87	177.93	<0.0001	
B^2^	115.37	1	115.37	161.81	<0.0001	
Residual	4.28	6	0.71			
Lack of fit	2.27	3	0.75	1.13	0.46	Not significant
Pure error	20.10	3	0.67			
Cor total	321.18	11				
(B) Response Y_2_						
Model	196.37	5	39.27	95.27	<0.0001	Significant
A-amount of carrier	22.81	1	22.81	55.35	0.0003	
B-amount of adsorbate	2.67	1	2.67	6.47	0.0439	
AB	7.84	1	7.84	19.02	0.0048	
A^2^	158.11	1	158.11	383.55	<0.0001	
B^2^	39.53	1	39.53	95.89	<0.0001	
Residual	2.47	6	0.41			
Lack of fit	1.88	3	0.63	3.19	0.18	Not significant
Pure error	0.59	3	0.20			
Cor total	198.84	11				

**TABLE 5:** (A) Fit statistics of response Y_1_ (B) Fit statistics of response Y_2_.

(A) Response Y_1_			
SD	0.84	R^2^	0.9867
Mean	86.02	Adjusted R^2^	0.9756
C.V.%	0.98	Predicted R^2^	0.9403
​	​	Adeq precision	33.20

(B) Response Y_2_			
SD	0.64	R^2^	0.9876
Mean	13.13	Adjusted R^2^	0.9772
C.V.%	4.89	Predicted R^2^	0.9016
​	​	Adeq precision	31.20

On analyzing the equation, it can be inferred that the amount of carrier and the amount of adsorbent increased; a progressive increase in %CDR at Q30 min takes place. PEG polymers are employed extensively because of their hydrophilicity, broad medication compatibility, low melting point, and low toxicity (Shah et al., 2015). When the amount of carrier (PEG 4000) is increased, there is an increase in %CDR as seen in Equation 3.1 and Figure 1A. Candesartan Cilexetil dissolves more quickly as a result of PEG 4000. When the PEG comes into contact with the dissolving liquid, it becomes hydrated. As a result, the drug is released, and the drug particles become soluble (Patel et al., 2021). The hydrophilic carrier PEG 4000 may be responsible for the solubilizing effect throughout the dissolving process. It may also reduce the interfacial tension between hydrophobic particles and the dissolution liquid, increasing the cumulative percentage of drug release (Patel and Mahajan, 2020).

**FIGURE 1:**
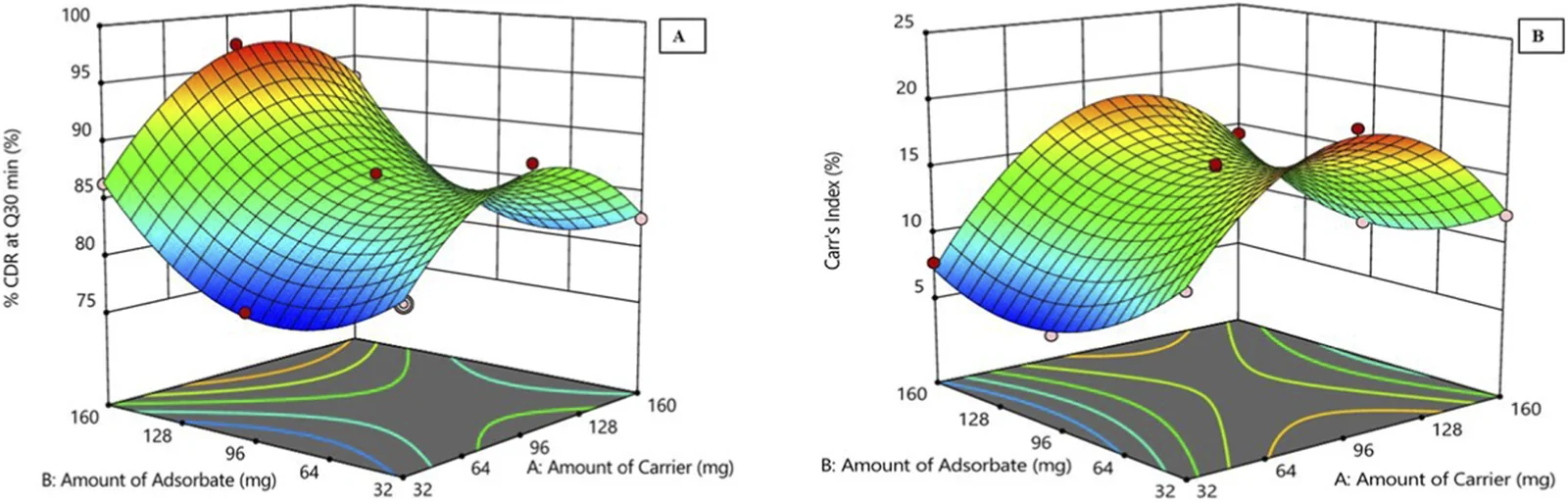
**(A)** 3D plot for %CDR at Q30 min **(B)** 3D plot for Carr’s index.

The effect of the adsorbate on %CDR showed that the higher the amount of adsorbate, the higher the %CDR, as seen in Equation 3.1 and Figure 1B (Patel et al., 2007). This is because Neusilin® US2 has numerous intraparticle and interparticle pores on its surface, making it a porous calcium silicate. Because of its high specific surface area (300 m^2^/g) and good adsorption capacity (3.2 mL/g), it is a valuable material for the adsorption of a high proportion of medications. When CC-SDAs are exposed to a dissolving solvent, the presence of Neusilin® US2 at the same time increases the effective surface area across which Candesartan Cilexetil is diffused. This speeds up drug desorption (Yadav et al., 2021).

#### 3.3.2 Data analysis of Carr’s index (Y_2_)

The Carr’s Index of the prepared formulations is found in the range between 5.12% ± 0.79% and 20.14% ± 0.47%, as shown in Table 3. The model’s F value of 95.27 indicated its significance (Table 4). To determine how each independent variable affected the outcome, an ANOVA was conducted (Table 4). In this case, the model terms A, B, AB, A^2^ and B^2^ are significant (p < 0.05). The F-value of 3.19 (p = 0.1830) indicates that the lack of fit is not significant. The correlation coefficient value (R^2^), which was determined to be 0.9876 (Table 5), indicated a reasonable fit. The adjusted R^2^ of 0.9772 (Table 5) was in reasonable agreement with the predicted R^2^ value of 0.9016. An adequate signal was indicated by an adequate precision value of 31.20 (>4) (Table 5).

$$\text{Carr}’s\text{ Index }\left(Y_{2}\right)=+15.05+1.95A-0.6667B+1.40\text{AB}-7.70A^{2}+3.85B^{2}$$ (3.2)

Equation 3.2 and the 3D response surface (Figure 1B) explained how each independent variable affects Carr’s index. An increase in Carr’s Index occurs when the amount of carrier is increased (Eraga et al., 2017). In other words, as the adsorbate increased, Carr’s Index was found to improve. This phenomenon is due to the adsorbate having the capability to improve effective surface area and improve porosity and tends to improve flow properties (Patel et al., 2007).

### 3.4 Optimization and validation

An overlay plot (Figure 2) with a design space based on the constraints was produced using DoE software. A checkpoint batch was chosen from the area of the design space where the recommended amounts of carrier and adsorbate were 41.47 mg and 159.36, respectively, while the expected values for the carr’s index and %CDR at Q30 min were 9.72% and 88.70%, respectively. By comparing the experimental values (actual) of the chosen batch with the predicted values of the dependent variables, the accuracy of the checkpoint batch (optimal formulation) was confirmed. The experimental %CDR at Q30 min is 90.41% ± 0.95%, and the Carr’s index is 9.86% ± 0.09%, respectively; this shows that the response surface model design for optimization has an excellent predictive ability, with an error of less than 5% for both responses (Table 6). As a result, the improved formulation was selected for further characterization studies.

**FIGURE 2:**
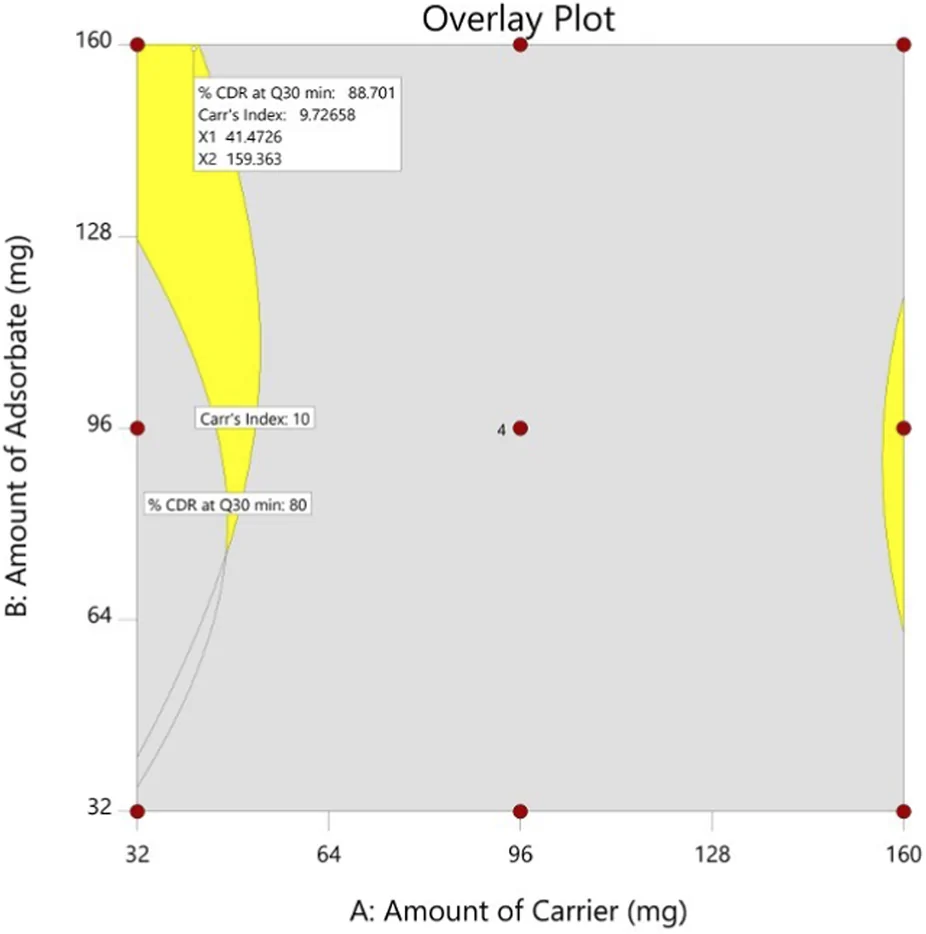
Overlay plot for response Y_1_ and Y_2_.

**TABLE 6:** Predicted and observed batch.

Parameter	Predicted	Observed	Error
Amount of carrier (PEG 4000) (X_1_)	41.47	​	​
Amount of adsorbate (NEUSILIN® US2) (X_2_)	159.36	​	​
Carr’s index (%)	9.72	9.86 ± 0.09	1.41%
% CDR (Q30 min)	88.70	90.41 ± 0.95	1.88%

(n = 3, ±SD).

The angle of repose, Hausner ratio, saturated solubility, and drug content of the optimized batch were found to be 26.00° ± 0.19°, 1.10 ± 0.16, 0.057 ± 0.005 mg/mL, and 97.27% ± 2.61%.

### 3.5 Compressibility and flowability

The Carr’s Index, Hausner’s Ratio, and Angle of Repose of the granules from batches B_1_-B_12_ were evaluated. The results ranged from 5.12% ± 0.79% to 20.14% ± 0.47% for Carr’s Index, 1.05 ± 1.54 to 1.25 ± 0.59 for Hausner’s Ratio, and 22.53° ± 2.99° to 38.71° ± 1.34° for the Angle of Repose, indicating fair flow properties. Neusilin® US2 is a porous, free-flowing carrier made up of spherical particles. It exhibits excellent flowability and high adsorptive capacity, enhancing the overall blend properties. The adsorption of the typically sticky solid dispersion onto Neusilin® US2 significantly improved the flow characteristics of the formulation (Yadav et al., 2021).

### 3.6 Fourier transform infrared spectroscopy (FTIR)

At 2940 cm^-1^ (-C-H stretching), Candesartan Cilexetil had a significant, strong bend along with the following distinct peaks. C-H stretching at 745 cm^-1^, N-N at 1032 cm^-1^, C-O at 1074 cm^-1^, C-N at 1240 cm^-1^, stretching (aromatic) at 1474 cm^-1^, N-H bending at 1546 cm^-1^, N-H stretching at 1611 cm^-1^, C=O stretching at 1751 cm^-1^ and C-H stretching at 2940 cm^-1^ (Gurunath et al., 2014). When C-O stretched at 1098 cm^-1^ and C-H stretched at several bands, Candesartan Cilexetil was created as SDA. The apex of SDA began to decline (Figure 3). This is because SDA forms intermolecular hydrogen bonds.

**FIGURE 3:**
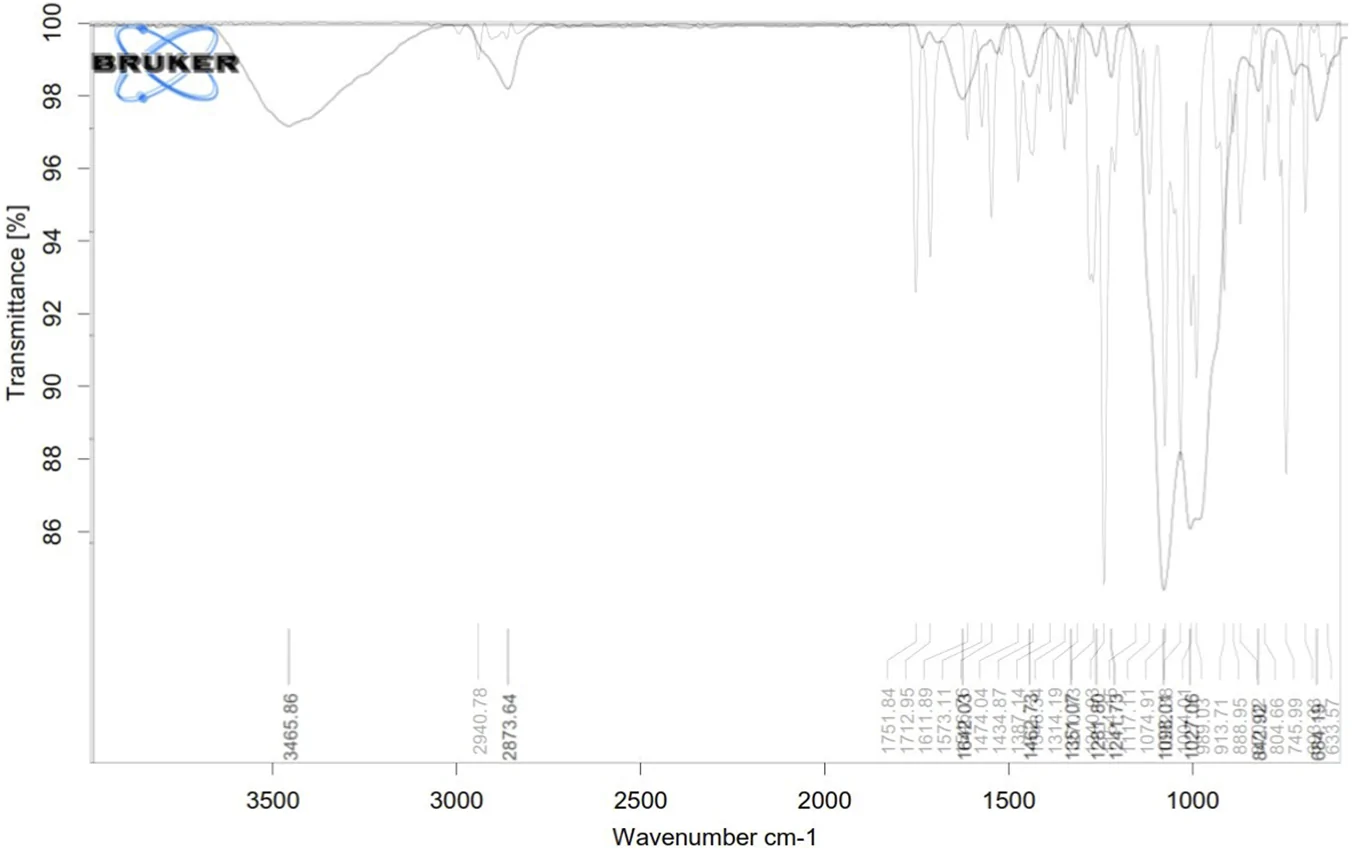
FTIR overlay of CC-SDAs on CC.

### 3.7 Differential scanning calorimetry (DSC)

Candesartan Cilexetil showed a single sharp endothermic peak at about 172.4 °C (Figure 4A), which matched its melting point and verified that it was crystalline. The drug’s molecular dispersion within the melted PEG 4000 matrix was responsible for the total elimination of the drug’s melting peak in the SDA (Figure 4B). Candesartan Cilexetil transition from a crystalline to an amorphous state and its molecular dispersion are important factors for improving dissolution (Lee et al., 2017).

**FIGURE 4:**
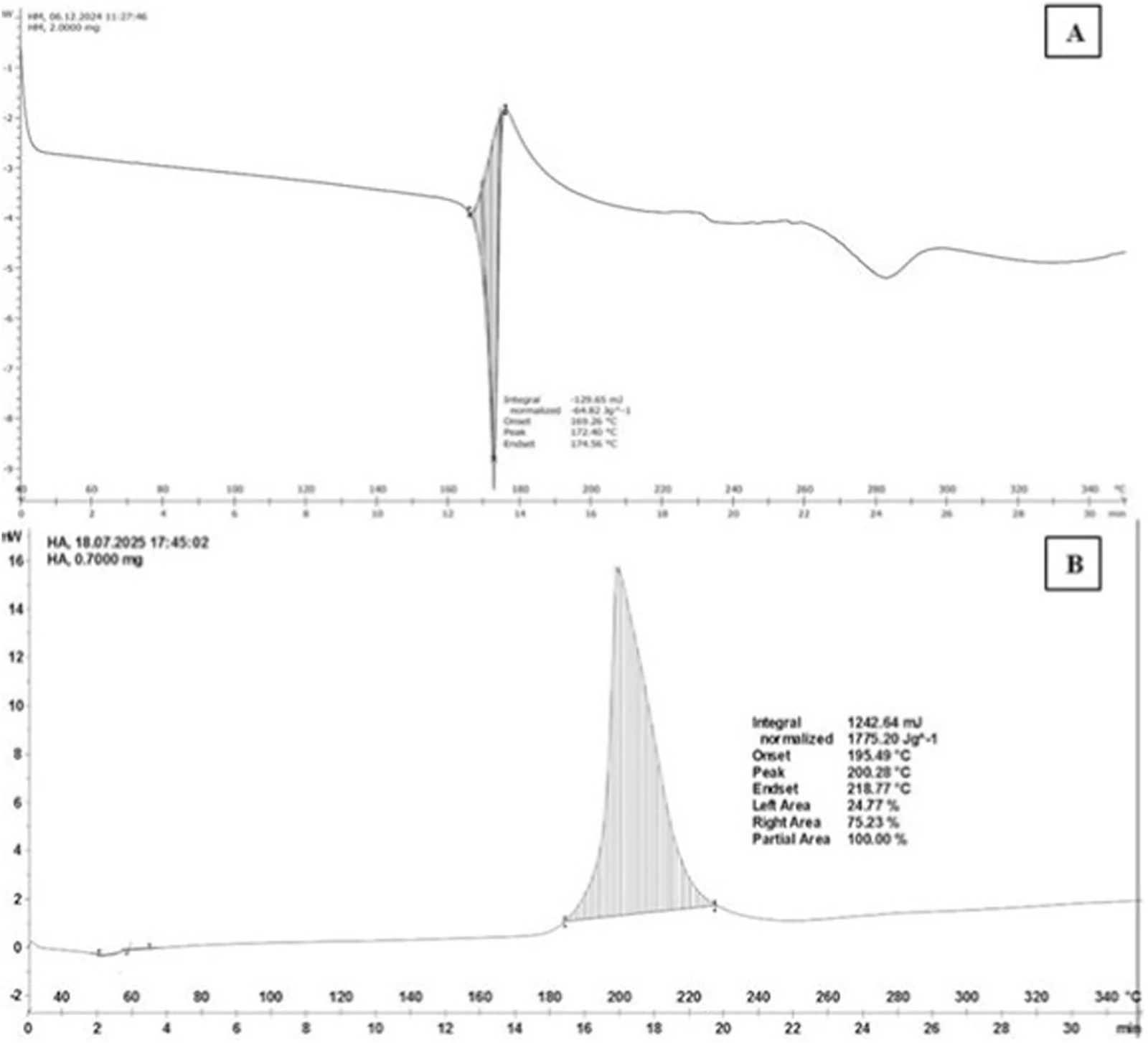
DSC of **(A)** CC **(B)** CC-SDAs.

### 3.8 Powder X-ray diffraction (XRD)

Candesartan Cilexetil showed sharp distinctive peaks at 2θ values of approximately 11°, 12°, 17°, 18°, 20°, 23°, and 25°, indicating its crystalline nature (Figure 5A). In the case of the SDA, disappearance of  Candesartan Cilexetil characteristic diffraction peaks indicating loss of drug crystallinity which is in agreement with DSC results (Figure 5B) (Dudhipala and Veerabrahma, 2016; Taokaew et al., 2020).

**FIGURE 5:**
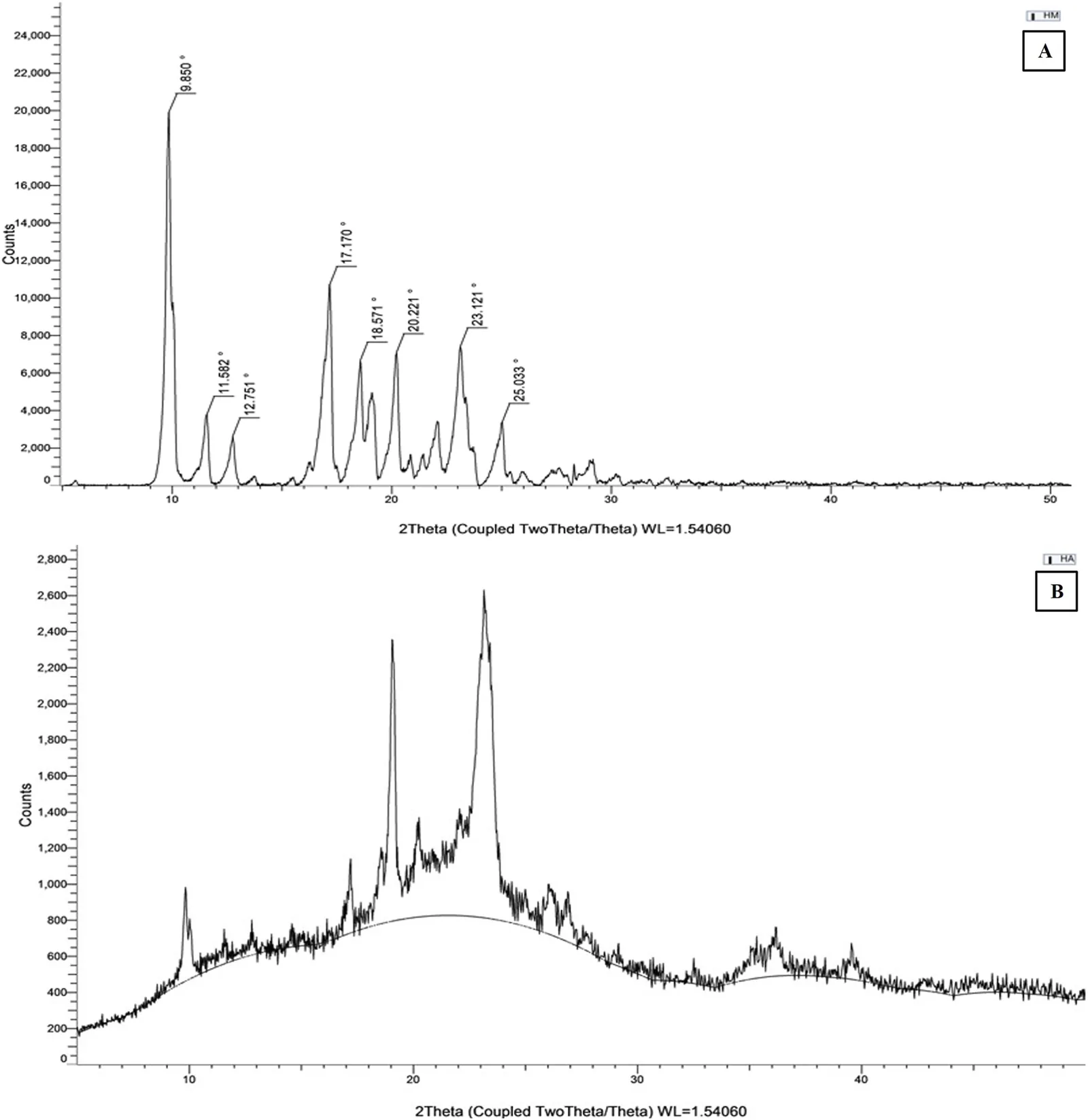
XRD of **(A)** CC **(B)** CC-SDAs.

### 3.9 Scanning electron microscopy (SEM)

SEM of Candesartan Cilexetil demonstrated that pure Candesartan Cilexetil (Figure 6A) has a crystalline nature with a rod-shaped crystal morphology. The surface of CC-SDA, on the other hand, was seen to be coated with agglomerates of adsorbates and a somewhat porous structure (Figure 6B). In comparison to the crystalline-like morphology seen in the SEM image of Candesartan Cilexetil, these morphological characteristics imply that SDA primarily occurs in an amorphous form, as demonstrated by its SEM image (Kumar and Suresh, 2019).

**FIGURE 6:**
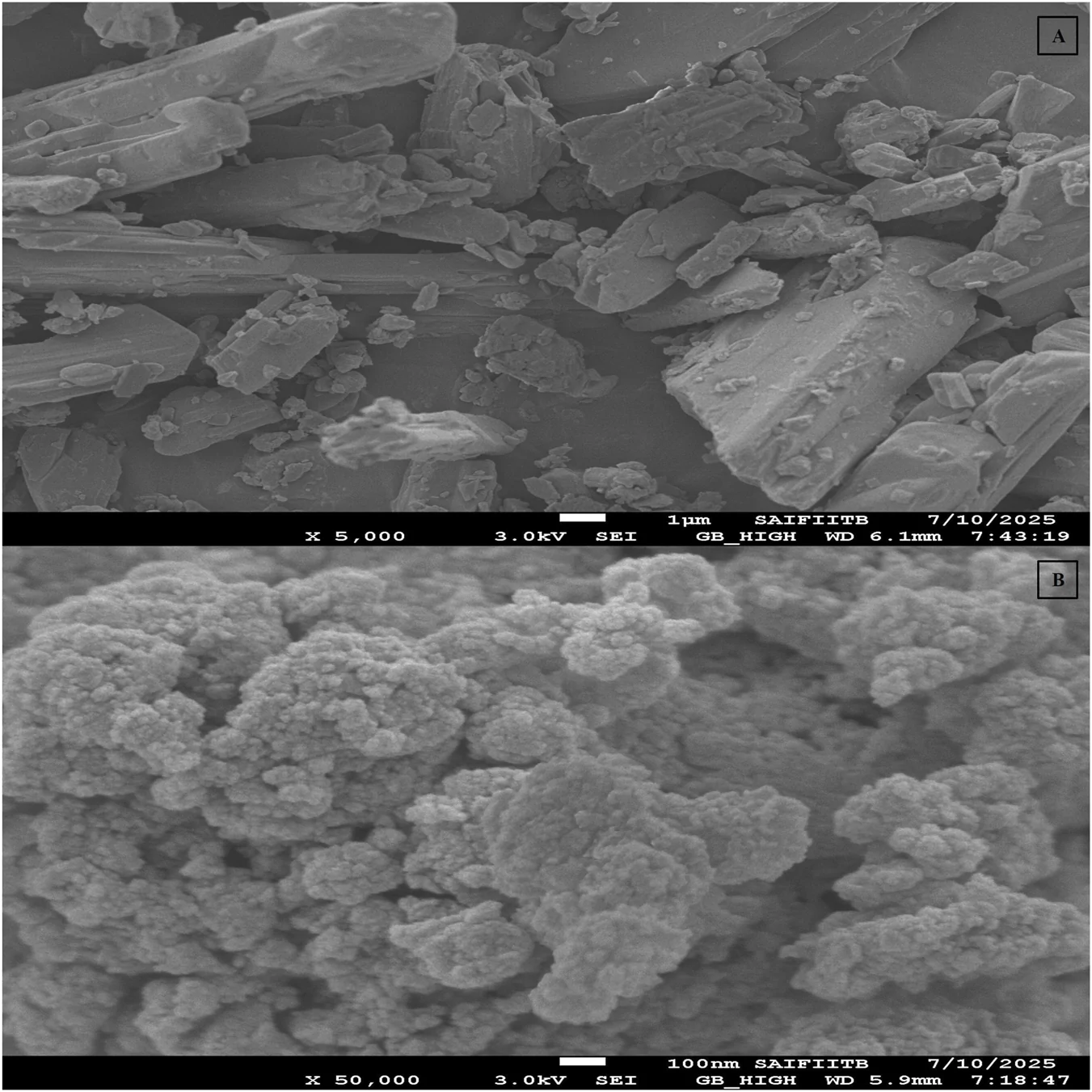
SEM of **(A)** CC **(B)** CC-SDAs.

### 3.10 Contact angle

Using the observation of sessile drop relaxation, a series of contact angle measurements was carried out to ascertain a link between surface drug concentration and wettability. The contact angle has two limits: 0° for total wetness and 180° for no wetting. Water was frequently used as the wetting solution in the test, despite the fact that the contact angle might change in different solutions. The contact angles of Candesartan Cilexetil and optimized CC-SDAs were displayed in Figures 7A,B. Due to its weak water solubility, Candesartan Cilexetil had a contact angle of 113.5°, whereas SDAs had a contact angle of 34.8°. When PEG 4000 was added, SDAs’ contact angle decreased, increasing their wettability (Lu et al., 2014).

**FIGURE 7:**
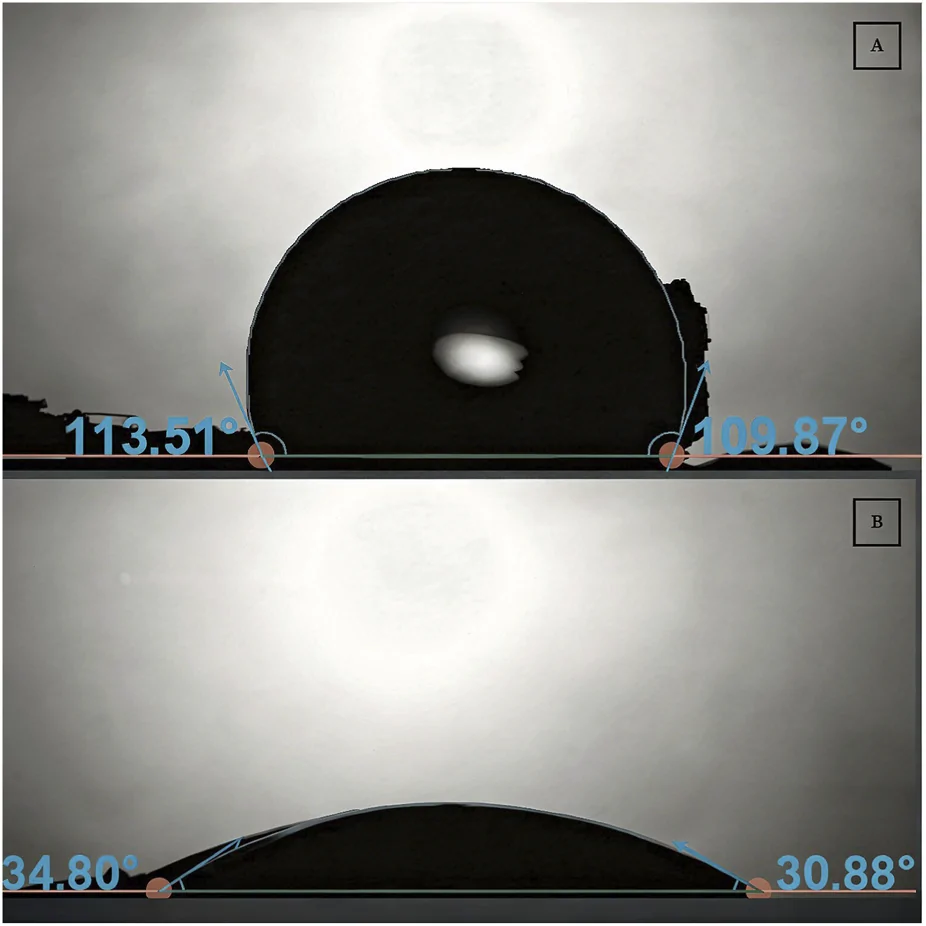
Contact angle by sessile drop relaxation of **(A)** CC **(B)** Optimized CC-SDA.

### 3.11 *In vitro* dissolution release

The paddle apparatus was used to perform the *in vitro* drug release of pure drug, CC-PM, and optimized SDAs (Figure 8; Table 7). The Q30 min values for pure drug, CC-PM and optimized CC-SDAs were 57.20% ± 3.78%, 69.51% ± 2.44%, and 90.41% ± 0.95%, respectively. After 60 min, 76.58% ± 2.80% of the pure drug, 90.20% ± 3.25% of the CC-PM SDA, and 98.07% ± 2.13% of the optimized CC-SDAs were released, respectively. When compared to the pure drug, there is somewhat higher dissolution of Candesartan Cilexetil from the CC-PM. This may be explained by the increased wettability and, hence, solubility brought about by the inclusion of the hydrophilic carrier PEG 4000. In addition, CC-SDA demonstrated a better Candesartan Cilexetil solubility than the Candesartan Cilexetil in its pure form. The presence of Candesartan Cilexetil in an amorphous state causes a greater dissolution of Candesartan Cilexetil from SDA in contrast to the PM and pure drug, where Candesartan Cilexetil exists in a crystalline state. Furthermore, a larger surface area would be provided by the adsorption of CC-SDAs onto Neusilin® US2, allowing for greater disintegration (Bajracharya et al., 2019).

**FIGURE 8:**
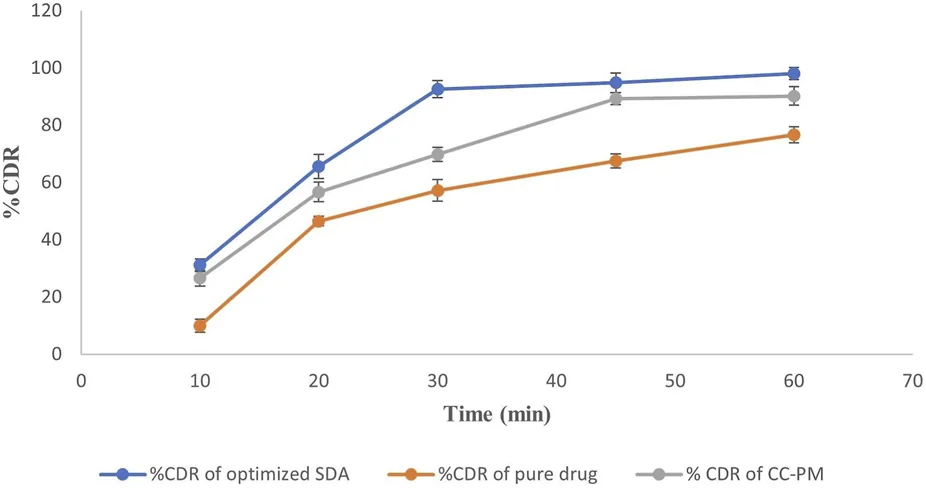
Cumulative drug release data of optimized CC-SDAs, pure drug, and CC-PM (n = 3, ±SD, P < 0.05, compared to pure drug and CC-PM).

**TABLE 7:** *In vitro* drug release of %CDR of pure drug, CC-PM, and %CDR of optimized SDA.

Time	%CDR of pure drug	%CDR of CC-PM	%CDR of optimized SDA
10 min	9.97 ± 2.27	26.52 ± 2.70	31.13 ± 2.19
20 min	46.51 ± 1.61	56.68 ± 3.36	65.59 ± 4.16
30 min	57.20 ± 3.78	69.51 ± 2.44	90.41 ± 0.95
45 min	67.51 ± 2.39	89.22 ± 2.11	94.81 ± 3.43
60 min	76.58 ± 2.80	90.20 ± 3.25	98.07 ± 2.13

(n = 3, ±SD).

When compared to the pure drug, our created formulation (CC-SDA-based capsules) showed a greater percentage of drug release at all time points. Increased *in vitro* dissolution could lead to an increase in vivo dissolution, which could result in a faster T_max_, higher C_max_, AUC, and bioavailability, as well as fewer side effects and therapeutic costs.

### 3.12 *In Vivo* pharmacokinetic study

Figure 9 and Table 8 display the pharmacokinetic information for the CC suspension, CC-PM, and CC-SDA suspension. The peak plasma concentration (Cmax) and the time to peak drug concentration (Tmax) were recorded using the generated plasma concentration vs. time profile. GraphPad Prism version 4 was used to calculate the area under the concentration-time curve (AUC_0-t_) using the linear trapezoidal method.

**FIGURE 9:**
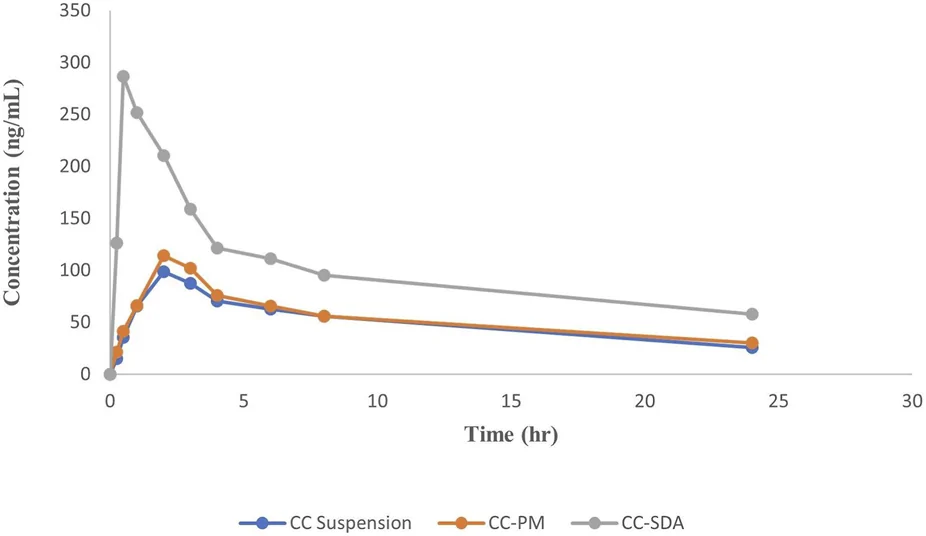
Plasma concentration-time plots of CC after oral administration of CC suspension, CC-PM, and CC-SDAs.

**TABLE 8:** Pharmacokinetic parameters of CC after oral administration of CC suspension, CC-PM, and CC-SDA suspension (3 mg/kg) in rats.

Parameters	CC suspension	CC-PM	CC-SDA suspension
C _max_ (ng/mL)	98.01 ± 0.40	114.3 ± 0.98	286.50 ± 1.00
T _max_ (h)	2.00	2.00	0.50
AUC_0-t_ (ng/mL*h)	1188.63 ± 2.60	1273.48 ± 1.68	2409.63 ± 2.20

(n = 3, ±SD, P < 0.05, compared to CC, suspension and CC-PM).

For CC-SDA suspension, the Tmax was 0.5 h; for CC-PM and CC suspension, it was 2.0 h. The CC-SDA suspension shows a quick rate of absorption in comparison to the CC suspension and CC-PM, which would probably cause a corresponding drop in the time to the beginning of the pharmacological effect. This is because the CC-SDAs in the GI tract increased the rate of Candesartan Cilexetil dissolution and solubility, enabling quick Candesartan Cilexetil absorption. At every time point, the plasma concentration levels of Candesartan Cilexetil were considerably greater (P < 0.05) for CC-SDA than for CC suspension and CC-PM. Cmax values were found to be 286.50 ± 1.00 ng/mL for CC-SDA, 114.3 ± 0.98 ng/mL for CC-PM, and 98.01 ± 0.40 ng/mL for CC suspension. Cmax values for CC-SDA suspension were 2.9 times higher than those for CC suspension, respectively, showing improved formulation release and ensuring systemic circulation and drug absorption. AUC_0-t_ of CC-SDA suspension was 2409.63 ± 2.20 ng/mL × h; for CC-PM, it was 1273.48 ± 1.68 ng/mL × h, whereas the CC suspension was 1188.63 ± 2.60 ng/mL × h, indicating a significant enhancement of bioavailability from the CC-SDA suspension. Higher Cmax and AUC_0-t_ obtained with the test formulation suggested improved oral bioavailability. *In vivo* absorption of the CC-SDA suspension was 2.0 times higher than that of the CC suspension, according to a comparison of the bioavailability of optimized CC-SDA suspension and the CC suspension.

The mixed-effects analysis showed significant effects of formulation (Table 9) (F (World Health Organization, 2025; Aparna et al., 2023) = 30,236.114, p = 9.764,647 × 10^-13), time (F (9, 54) = 31,900.100, p = 2.952,436 × 10^-97), and the formulation × time interaction (F (18, 54) = 10,572.728, p = 3.950,712 × 10^-89). Given the significant interaction, Holm-adjusted *post hoc* comparisons were interpreted at each time point. SDA produced significantly higher concentrations than both PM and suspension at every post-dose time point (all Holm-adjusted p ≤ 2.026265 × 10^-9). PM was significantly higher than suspension at 0.25, 0.5, 2, 3, 4, 6, and 24 h (Holm-adjusted p values from 5.963,869 × 10^-7 to 0.006360), but not at 1 h (p = 0.744,273) or 8 h (p = 1.000000). At baseline, no formulation differences were present (all p = 1.000000).

**TABLE 9:** Omnibus mixed-effects tests.

Effect	Numerator df	Denominator df	F	Exact p-value
Formulation	2	6	30,236.114	9.764647e-13
Time	9	54	31,900.100	2.952436e-97
Formulation × time	18	54	10,572.728	3.950712e-89

### 3.13 Stability studies

The flow characteristics of optimized CC-SDAs (Table 10), such as angle of repose, Carr’s index, Hausner’s ratio, saturated solubility, and %CDR at Q30 min, did not significantly change from original values, even after 6 months of storage at 40 °C ± 2 °C/75% ± 5% RH. Taken together, these results simply indicate that optimized CC-SDAs are stable over the storage period.

**TABLE 10:** Stability study of optimized CC-SDAs.

Storage temperature	Angle of repose (°)	Carr’s index (%)	Hausner’s ratio	Saturated solubility (mg/mL)	%CDR at Q30 min
Initial	26.00 ± 0.19	9.86 ± 0.09	1.10 ± 0.16	0.057 ± 0.005	90.41 ± 0.95
40 °C ± 2 °C/75% ± 5%	24.15 ± 0.15	8.69 ± 0.37	1.09 ± 0.05	0.064 ± 0.070	89.14 ± 3.00

## 4 Conclusion

CC-SDAs were formulated by the fusion process using PEG 4000 as a carrier and Neusilin® US2 as an adsorbate. The formulation was optimized using a 3^2^ factorial design. Neusilin US2, a large surface area adsorbent; PEG 4000, a hydrophilic carrier; and Candesartan Cilexetil, an insoluble drug, made up the ternary system that was crushed into the capsule without any of the typical processing issues that arise when using capsule solid dispersions. Characterization investigations confirmed that the CC-SDA shows immediate drug release, good flow characteristics, and loss of crystallinity. The dissolution profile of CC-SDAs was found to be higher than that of the pure drug, which shows improved dissolution. The improved dissolution may be caused by hydrogen bonding between the Candesartan Cilexetil and Neusilin® US2, micellar solubilization of the drug in the carrier, increased wettability, and decreased crystallinity. This technique’s main advantage is that it prevents the amorphous form from returning to the crystalline form. The improved formulation met the USP requirement of more than 85% drug release within 30 min. The combined action of SD and increased surface area caused by adsorption may be responsible for the enhanced flow characteristics, solubility, and dissolution of the CC-SDAs formulations. An *in vivo* pharmacokinetic analysis using CC-SDA suspension in male Wistar rats showed that Candesartan Cilexetil had a faster action and greater bioavailability than CC suspension. The outcome shows that SDA is a new and promising method for improving CC dissolution and can be applied to the creation of an appropriate solid dosage form for market release.

## Data Availability

The original contributions presented in the study are included in the article/supplementary material, further inquiries can be directed to the corresponding author.
